# Incidental Findings: The Importance of Pretest Counseling

**DOI:** 10.15844/pedneurbriefs-29-12-2

**Published:** 2015-12

**Authors:** Kathryn M. Buchtel, Elizabeth A. Leeth

**Affiliations:** 1 Graduate Program in Genetic Counseling, Northwestern University, Chicago, IL; 2 Department of Pathology, Ann & Robert H. Lurie Children's Hospital of Chicago, Chicago, IL

**Keywords:** aCGH, ethical issues, incidental findings, pre-test information

## Abstract

Researchers at the University of Bourgogne in Dijon, France surveyed French geneticists who were members of the “Association Français des Généticiens” on incidental findings (IF) found on array-based comparative genomic hybridization (aCGH) technology retrospectively over a seven-year period.

Researchers at the University of Bourgogne in Dijon, France surveyed French geneticists who were members of the “Association Français des Généticiens” on incidental findings (IF) found on array-based comparative genomic hybridization (aCGH) technology retrospectively over a seven-year period. Data analyzed on 65 cases had IF for autosomal dominant conditions with a range of penetrance, X-linked conditions, and heterozygous carriers of an autosomal recessive condition. Overall, 79% were classified as pathogenic or likely pathogenic, and as a variant of uncertain significance in 21% of cases. Of the 65 cases, all but four warranted some type of preventive care, change of management, or genetic counseling. These four results were not returned to the patients. One was chosen to not be returned due to the lack of pretest counseling on IF. The respondents reported difficulty in returning IF to patients 29% of the time, especially in the case where a possibility of an IF was not anticipated by the clinician. Only 21 (48%) reported using a consent process and pretest counseling on the possibility of IF. [1]

COMMENTARY. The availability of advanced genetic technology enables analyses for multiple disorders to be done concurrently. This has led to the incidental finding of medical information unrelated to the clinical indication for testing. The struggles surrounding incidental or secondary findings (IF/SF) are not new, however, this paper exemplifies the continued dilemmas surrounding the informed consent process and the lack of clear direction for providers in disclosing IF information to patients.

The ACMG has addressed informed consent for IF/SF [2]. However, these processes remain inconsistent for IF/SF encountered via aCGH and large NGS panels which test for groups of disorders and are not limited to analysis of only phenotypically indicated diseases. A recent review of large NGS epilepsy panels highlighted the importance of knowing the content of these panels so that accurate pretest information can be provided to improve the informed consent process [3]. While it is unrealistic to provide counseling on every IF possible, it is realistic to provide anticipatory guidance as to the range of impact of these IF.

The reporting of difficulty in the disclosure of IF by the providers, especially when not anticipated, validates previous recommendations that all practitioners anticipate and plan for IF in pretest discussions with patients [4]. An approach where IF are expected would normalize the scenario for patients and providers so that a shared-decision-making process can be utilized to promote the delivery and receiving of results. The need to delineate what is required to provide accurate information to guide these discussions has been recognized [5]. Such resources will prove crucial for adequate informed consent and understanding for both clinicians and patients. With this endpoint in mind, further recommendations, guidelines, education, and resources for pretest counseling and consent requirements surrounding IF/SF need to be formalized to ensure consistent practice.
